# Geomagnetic Anomaly in the Growth Response of Peat Moss *Sphagnum riparium* to Temperature

**DOI:** 10.3390/plants13010048

**Published:** 2023-12-22

**Authors:** Victor L. Mironov

**Affiliations:** Institute of Biology of the Karelian Research, Centre of the Russian Academy of Sciences, Pushkinskaya St. 11, 185910 Petrozavodsk, Russia; vict.mironoff@yandex.ru

**Keywords:** boreal peatland, peat moss, growth monitoring, geotropic curvature method, temperature, geomagnetic K_p_ index

## Abstract

Temperature plays an essential role in a plant’s life. The current investigation reveals that photoreceptors, whose activity is affected by the geomagnetic field, are a critical element of its perception. This knowledge suggests that plants’ responses to temperature could shift in different geomagnetic conditions. To test this hypothesis, we studied the change in the growth response of the peat moss *Sphagnum riparium* to temperature with a gradual increase in the geomagnetic K_p_ index. Growth data for this species were collected from Karelian mires by detailed monitoring over eight full growing seasons. The growth of 209,490 shoots was measured and 1439 growth rates were obtained for this period. The analysis showed a strong positive dependence of sphagnum growth on temperature (r = 0.58; n = 1439; P = 1.7 × 10^−119^), which is strongest in the K_p_ range from 0.87 to 1.61 (r = 0.65; n = 464; P = 4.5 × 10^−58^). This K_p_ interval is clearer after removing the seasonal contributions from the growth rate and temperature and is preserved when diurnal temperature is used. Our results are consistent with the hypothesis and show the unknown contribution of the geomagnetic field to the temperature responses of plants.

## 1. Introduction

Temperature is crucial for controlling most plant processes [1]. Growth and development, hierarchy in communities, and, ultimately, the distribution areas of plants depend on how accurately they perceive and then respond to temperature changes. Such an important role of temperature largely follows from the Van’t Hoff rule, according to which an increase in temperature of 10 °C accelerates the chemical reactions underlying living processes by about 2–4 times [2]. Besides this mechanism, plants can rely on temperature-dependent alterations in membrane fluidity, protein conformation, cytoskeleton depolymerization, and some metabolic reactions [3]. However, some photoreceptors have recently been found to be extensively utilized by plants for optimal temperature sensing [4].

One of them is phytochrome B, best known as a red/far red light photoreceptor, which is involved in photoperiodic processes [5] and some temperature-sensitive plant responses [6,7]. The spontaneous reversion of active P_fr_ to inactive P_r_ in phytochrome B has a strong temperature dependence, which makes it a temperature-sensitive sensor [8,9]. The other is phototropin, known as the blue light photoreceptor in plants, which is involved, for example, in shoot phototropism and chloroplast movement [10]. The lifetime of a photoactivated phototropin is also strongly dependent on temperature, so it can perceive temperature changes [11]. Both of these photoreceptors in plants cross-talk with blue and UV-A light photoreceptors, cryptochromes [12,13], which are considered important components of the biological perception of the geomagnetic field [14].

The geomagnetic field is an extremely weak but essential component of the natural environment to which plants have been exposed throughout their history. Its intensity is commonly 25–65 μT at the level of the Earth’s surface, while fluctuations in solar activity cause variations of up to 10% relative to these values [15]. A growing number of studies show that plants can sense magnetic fields and fit their processes to them [16,17,18,19]. In particular, flowering, photosynthesis, root and shoot growth, mineral nutrition, and even key events in plant evolution appear to be related to magnetic fields [16,18]. The primary source of the magnetic sense of plants has not yet been discovered. On the one hand, the participation of cryptochromes in the processes of magnetoreception has been convincingly shown [14]. The starting point for this is the formation of a radical pair, which is a natural magnetosensor, after the irradiation of a cryptochrome with blue light [20,21]. On the other hand, evidence of light-independent sensitivity of plants to a magnetic field has accumulated in recent years [22,23,24], which makes the existence of alternative magnetosensors possible.

The involvement of cryptochromes, which cross-talk with phytochrome B [12] and phototropin [13], in magnetic sensing suggests that variations in the geomagnetic field may modulate signaling of the latter. Experimental confirmation of this hypothesis has begun to accumulate in recent decades [22,23,25,26]. However, there is still no data on whether the geomagnetic field modulates the temperature signaling of these receptors, which should ultimately be reflected in the temperature-dependent responses of plants. One of the simplest indicators of such modulation can be a shift in the temperature growth response with a change in the geomagnetic K_p_ index (the description of K_p_ index is presented in Section 2.4), a key indicator of the geomagnetic field [27,28]. It can exist as a weak increase and decrease in the temperature growth response, which are carefully masked by the continuously varying geomagnetic field.

To resolve this uncertainty, here, we include data from detailed growth monitoring of the peat moss *Sphagnum riparium* Ångstr. (Sphagnaceae, Bryophyta), carried out in the mires of Karelia (Russia) since 2015. At present, the study covered eight full growth periods of this species, during which the growth of 209,490 shoots was measured with a predominant interval of 2 days and 1439 growth rates were obtained. We have previously shown that temperature is the strongest environmental growth factor for *S. riparium* [29], but the current amount of data allows us to explore previously unknown aspects of its influence. In particular, the continuous action of temperature and the geomagnetic field on growing moss makes it possible to trace in detail how its temperature growth response changes along the K_p_ gradient.

## 2. Materials and Methods

### 2.1. Study Area

The study area is located in the middle taiga of southern Karelia (Russia). The study was carried out in the vicinity of the Petrozavodsk city, where in different years, two mires were research sites. The characteristics of these mires are presented in detail in previous papers [29,30,31,32].

The first study site is a mire with an area of 0.12 ha (N 61°51′14″; E 34°10′51″; 50 m a.s.l.) in 2015–2018. It is surrounded by a spruce forest, and its vegetation cover is represented by willow–sedge–sphagnum and sedge–sphagnum minerotrophic plant communities. Peat depth here is 30–80 cm, mire water level is +2–20 cm, water salinity is 14–54 mg L^−1^, and pH is 4.6–6.8. Sphagnum mosses form a continuous, undisturbed mat on almost the entire surface of the mire. Among them, *Sphagnum riparium* covers more than 90%, and *S. squarrosum*, *S. divinum*, *S. centrale*, *S. fallax*, and *S. angustifolium* covers up to 7% of the surface. Among the vascular plants are *Salix phylicifolia*, *Equisetum fluviatile*, *Calamagrostis canescens*, *Comarum palustre*, *Carex rostrata*, *Calla palustris*, and *Typha latifolia*, which cover from 5 to 30% of the surface. The study in this mire was terminated in 2018 due to reclamation.

The mire near Dennaya Lamba (N 61°44′39″; E 34°16′05″; 160 m a.s.l.), which is a complex of dry drained areas and wet draining ditches, was selected for continuation of the study after 2018. The dry drained areas are covered with pine–shrub–sphagnum communities dominated by shrubs *Andromeda polyfolia*, *Ledum palustre*, *Vaccinium vitis-ideae*, *Calluna vulgaris*, *Rubus chamaemorus*, and peat mosses *Sphagnum fuscum*, *S. angustifolium*, *S. capillifolium*, and *S. divinum*. The study was carried out in wet draining ditches, in which the wetting conditions are similar to the previous mire. The ditches are 150–200 cm wide, 400–600 m long, 0.5–1.5 m deep, mire water level 0–-15 cm, water salinity 45–156 mg L^−1^, and pH 4.3–6.5. The plant cover of the ditches is represented by a continuous mat of *Sphagnum riparium* with a rare admixture of *S. fallax* and *S. angustifolium*, as well as *Carex rostrata* and *C. magellanica* sedges.

### 2.2. Object of Study

Peat moss *Sphagnum riparium* Ångstr. (Sphagnaceae, Bryophyta) was the object of study. It is a large dioecious species with a circumpolar range in Europe, Asia, and North America [33]. It is widespread in open flooded mires, drainage ditches of mires, as well as in re-flooded wetlands within the study area. It has a ruderal growth strategy in these habitats [34], so shoots can grow over 40 cm in a season [35,36]. The high growth rate and the existence of shoots in the form of extended mats make this species a convenient model for detailed growth monitoring. In addition, *S. riparium* does not tend to dry out during its growth period, since its habitats are commonly well watered, so an important advantage when using it is the detection of the influence of environmental factors during the full growth period.

### 2.3. Growth Monitoring

The study was conducted from 2015 to 2022, during which time eight full growth periods of *S. riparium* were covered. Shoot growth measurements began immediately after the sphagnum mat thawed (commonly in the second half of April) and continued until it was frozen (commonly in the second half of October). They were carried out using the original geotropic curvature method [36]. The growth monitoring technique has been described in detail in previous papers [29,30,31,32].

**Figure 1:**
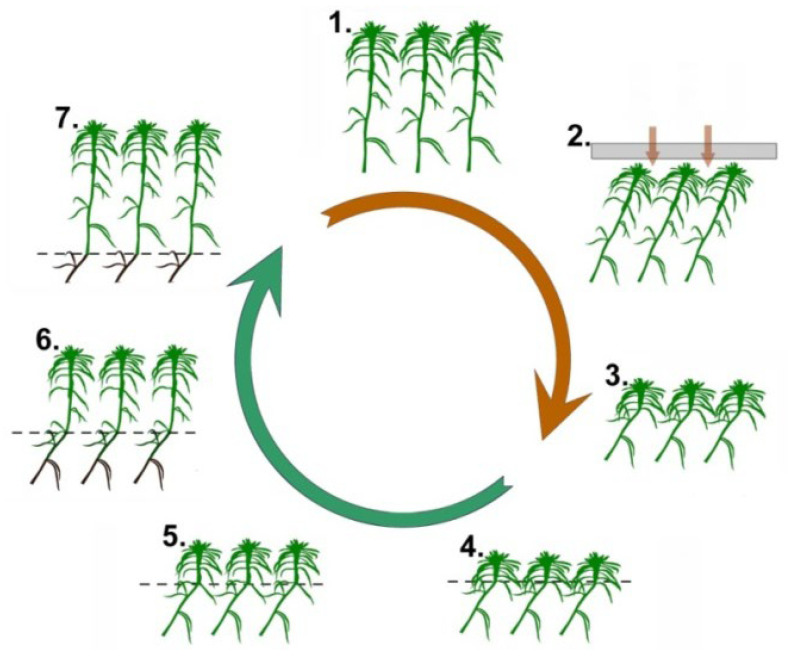
Growth monitoring of *S. riparium* using geotropic curvatures. The brown arrow (stages 1–3) reflects the induction of geotropic curvatures. It is based on the deviation of initially upright shoots (stage 1) from the vertical under snow or artificial load (stage 2). Geotropic curvatures are formed as natural growth markers on inclined shoots (stage 3). Subsequently, we carefully monitor and document the growth of shoots from these curvatures (green arrow, stages 4–7).

It is based on the use of nival and artificial geotropic curvatures formed in response to snow and artificial indentation of the sphagnum cover (Figure 1). Nival curvatures were used immediately after the spring thaw, as they are ubiquitous on the stems at that time. Artificial curvatures were used to reduce the random measurement errors when shoots were elongated by about 10 cm. They were induced by short-term indentation of a sphagnum mat with a plywood sheet. As a result, after 1–3 days, artificial curvatures appeared on the stems, which served as new growth markers for us. The induction of artificial curvatures was repeated as the shoots lengthened, mostly 3 to 6 times per season.

Growth monitoring was carried out on a series of sample plots. In different years, from 3 to 13 sample plots were used with sizes ranging from 3 × 3 m^2^ in the first mire to 5 × 1 m^2^ in the draining ditches of the second mire. Sample plots were established every year on an intact sphagnum mat with up to 15% coverage of vascular plants. Samples of shoots (commonly 30–60 shoots) from pieces of sphagnum mat about 100 cm^2^ were sequentially taken from each sample plot (commonly 68–102 sampling events per season). Each subsequent sampling was performed from an intact sphagnum mat with a several centimeters indent from the previous sampling site. The interval between the sampling events differed in different years, but for most years, it was 2 days. Based on the difference in growth at the beginning and end of each interval, we consistently obtained patterns of growth rates in each sample plot. Patterns for the whole mire area were obtained by averaging the corresponding growth rates over the sample plots.

The basic growth monitoring parameters in different years are summarized in Table 1. We measured the growth of 209,490 shoots and obtained 9959 growth rates on the sample plots during the monitoring period. These data provided the basis for the 1439 growth rates for the whole mire area that we used in the analysis.

### 2.4. Sources of Geomagnetic K_p_ Index and Temperature Data

The geomagnetic K_p_ index is one of the most common indicators of the geomagnetic field [27,28]. It is based on K indices determined at 13 reference magnetic observatories in the subauroral belt of the Earth. Each K index is the maximum observatory-specific deviation of the geomagnetic field from the norm during a 3 h interval, for which a value is assigned from 0 (calm geomagnetic field) to 9 (extremely disturbed geomagnetic field). The data source for the K_p_ index is Helmholtz Center Potsdam GFZ German Research Center for Geosciences, whose data are freely available at https://www.gfz-potsdam.de/en/section/geomagnetism/data-products-services/geomagnetic-kp-index (accessed on 10 April 2023).

The values of air temperature were obtained at the Petrozavodsk meteorological station (WMO ID 22820), located 4.5 and 8.2 km from the first and second mires. In the analysis in Section 3.5, we additionally use data on soil surface temperature at this weather station. The soil near the weather station is sandy and loamy, with a high content of small stones and pebbles.The soil surface is covered with a dense cover of herbaceous plants. The temperature data source was the AISORI database, the data of which are available at the link: http://aisori-m.meteo.ru/waisori/ (accessed on 10 April 2023).

**Figure 2:**
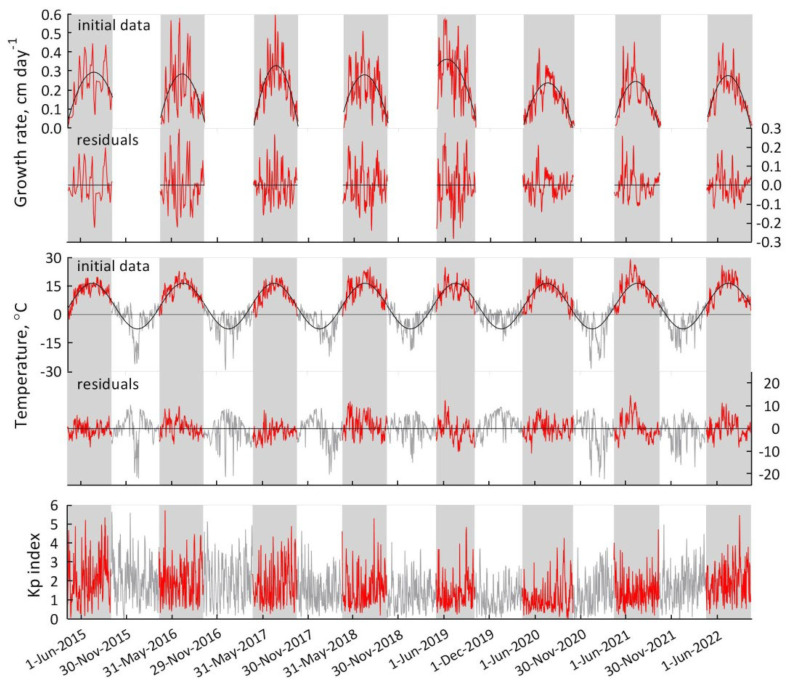
Growth rate of *S. riparium*, temperature, and geomagnetic K_p_ index for the period under study. The growth rate and temperature patterns show a polynomial and harmonic trend, respectively. The data included in the analysis are highlighted in color.

In the analysis, we used the K_p_ index and temperature data from the day prior to the growth rate to preserve causality. The corresponding patterns of *S. riparium* growth rate, temperature, and K_p_ index over the study period are shown in Figure 2.

### 2.5. Data Preparation and Analysis

The preparation of *S. riparium* growth rate patterns for analysis consisted of reducing the random variation caused by extrapolating the same growth rate value for each day of the observation interval. To do this, the patterns were filtered with a simple 3-day moving average [29], after which they were used in further analysis.

First, using the Pearson correlation coefficient and linear regression, the response of all growth rate values to temperature and the geomagnetic K_p_ index was estimated.

Then, using a sliding window correlation, the change in the growth response to temperature along the gradient of the K_p_ index was analyzed. To do this, all paired values of the growth rate and temperature were sorted in order of increasing K_p_ index. After that, in a sliding window of 200 values, the correlation coefficients between growth rate and temperature were calculated. The values of the K_p_ index were calculated in the corresponding window. As a result, a pattern was obtained, in which each value of K_p_ corresponds to a correlation coefficient between growth rate and temperature. This principle was used in two subsequent analyses.

For a better understanding of the issue, the change in the growth response to local temperature fluctuations along the K_p_ gradient was estimated. This analysis implied elimination of seasonal trends from both analyzed patterns. The second-order polynomial trend induced by the seasonal temperature course was removed from the growth rate pattern [29]. The harmonic trend created by the annual cycle was removed from the temperature pattern. This trend was built using the sum of sinusoids model. A sinusoid with a period of 365.3 days was taken as its model, which best approximates the temperature data by R^2^. After detrending, according to the principle described above, a sliding window correlation was carried out between the residuals of the growth rate and temperature.

In conclusion, the differences in the growth response to the diurnal temperature at 00:00, 03:00, 06:00, 09:00, 12:00, 15:00, 18:00 and 21:00 along the K_p_ index gradient were studied. For each of these hours, according to the principle described above, a sliding window correlation was made between the growth rate and temperature.

Data preparation and analysis were carried out using the freely available PAST 4.11 software.

## 3. Results

### 3.1. Growth Response to Temperature

**Figure 3:**
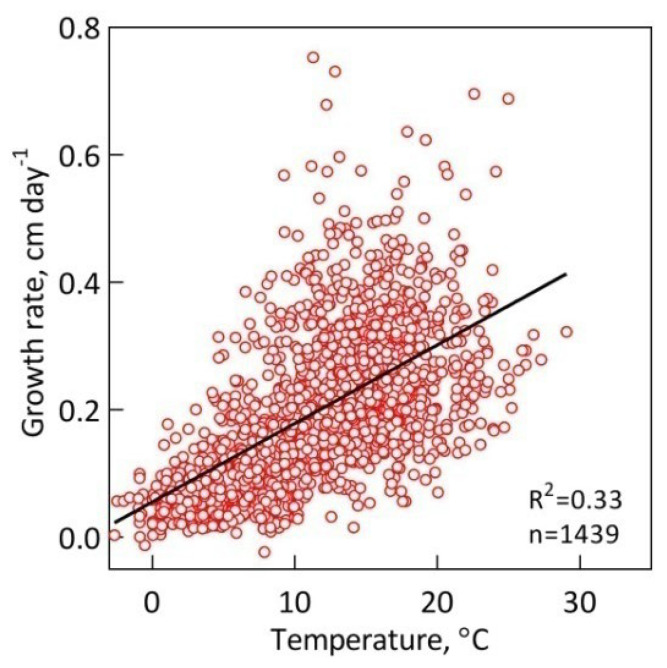
Growth response of *S. riparium* to temperature.

Initially, we analyzed the dependence of all *S. riparium* growth rates on temperature (Figure 3). As anticipated, temperature was found to have a strong positive impact on the Sphagnum growth rate (r = 0.58; n = 1439; P = 1.7 × 10^−119^). This effect is also clearly visible in Figure 2, where the growth rate pattern coincides with the seasonal course of temperature.

### 3.2. Growth Response to the Geomagnetic K_p_ Index

**Figure 4:**
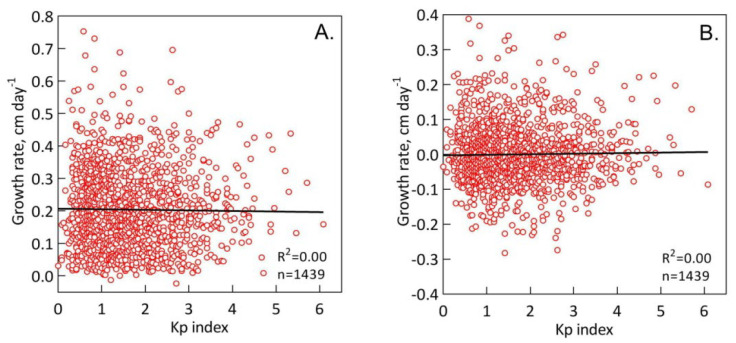
Growth response of *S. riparium* to the geomagnetic K_p_ index. The dependence of the growth rate (**A**) and its residuals (**B**) on the K_p_ index is presented.

We also assessed the dependence of all *S. riparium* growth rates (and its residuals) on the geomagnetic K_p_ index (Figure 4). In contrast to temperature, there was no significant response of growth rates (r = 5.4 × 10^−6^; n = 1439; P = 0.99) and its residuals (r = 0.03; n = 1439; P = 0.20) to the K_p_ index.

### 3.3. Change in Growth Response to Temperature along the K_p_ Gradient

**Figure 5:**
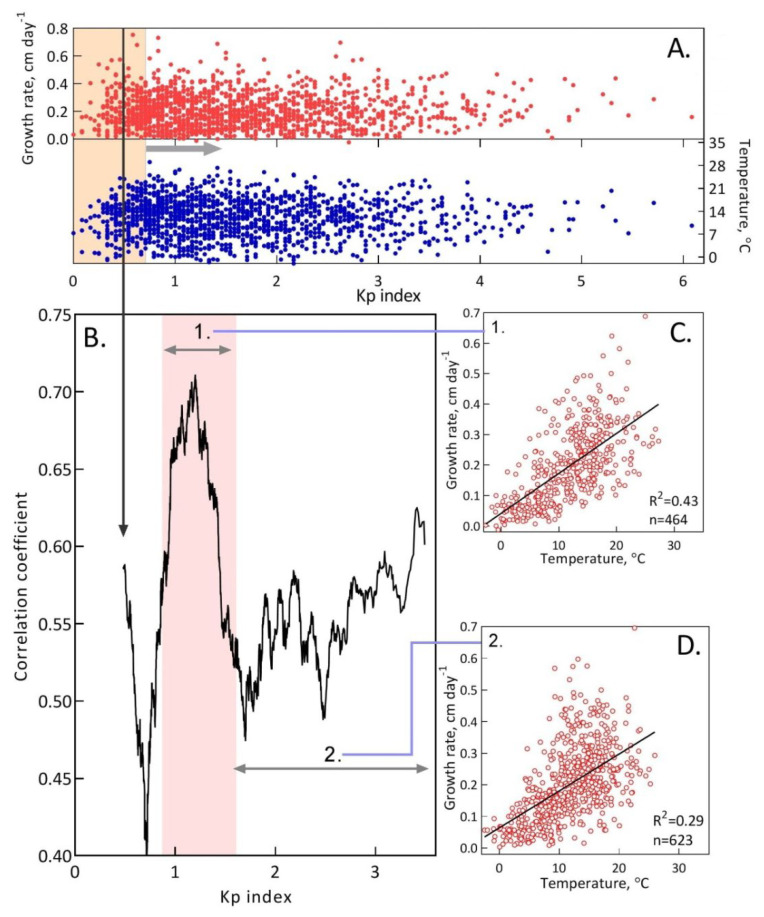
Change in the growth response of *S. riparium* to temperature along the K_p_ gradient. (**A**) Values of growth rate and temperature, sorted by the gradient of the K_p_ index. (**B**) Pattern of sliding window correlation coefficients. To calculate these correlation coefficients, here, we used a sliding window of 200 values (section (**A**) highlights an example window for the first coefficient). In this window, we calculated the average K_p_ (in section A, this is shown by a bold vertical line turning into an arrow) and the corresponding correlation coefficient. The K_p_ interval from 0.87 to 1.61 is highlighted in color. (**C**,**D**) Examples of the growth response to temperature at different ranges of the K_p_ indices (these ranges are shown by arrows in section (**B**)).

We then examined the modulating effect of the geomagnetic K_p_ index on the temperature growth response of *S. riparium*. The sliding window correlation between the growth rate and temperature along the K_p_ gradient showed a change in the temperature growth response along the K_p_ gradient (Figure 5). Here, we can distinguish three fragments with different growth responses to temperature.

The first is typical for a K_p_ index less than 0.87 and is generally characterized by a weakened growth response to temperature (r = 0.53; n = 352; P = 2.2 × 10^−26^), which reaches a minimum at K_p_ = 0.71. The second fragment, where we see an unusually robust growth response to temperature, is typical for K_p_ from 0.87 to 1.61. The correlation coefficients here exhibit a sharp increase, peak at K_p_ = 1.20, and then exhibit a decrease in this range. The correlation coefficient between all values in this range is r = 0.65 (n = 464; P = 4.5 × 10^−58^). Based on the total duration of our monitoring, we see that approximately one-third (32.2%) of the days within the Sphagnum growth exhibit favorable geomagnetic conditions for growth response to temperature. The third fragment is characterized by a generally weakened (r = 0.54; n = 623; P = 1.7 × 10^−48^) but gradually increasing growth response to temperature.

### 3.4. Change in Growth Response to the Minor Temperature Fluctuations along the K_p_ Gradient

**Figure 6:**
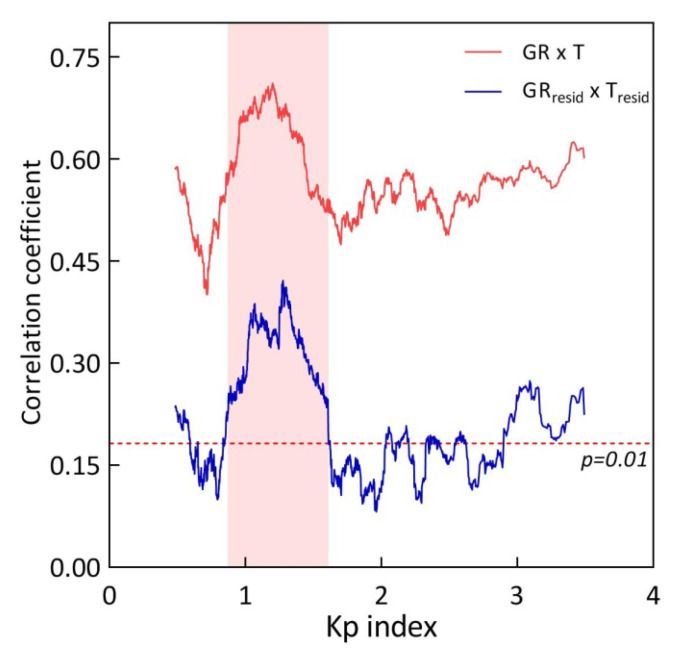
Change in the growth response of *S. riparium* to the local temperature fluctuations along the K_p_ gradient. Sliding window correlation coefficients calculated for (1) GR × T—growth rate and temperature (initial pattern, taken from Figure 5); (2) GR_resid_ × GR_resid_—growth rate residuals and temperature residuals. The K_p_ interval from 0.87 to 1.61 is highlighted in color.

Additionally, we evaluated the change in the growth response of *S. riparium* to the minor temperature fluctuations along the K_p_ gradient (Figure 6). Their simplest model was temperature residuals extracted from the average daily temperature pattern. Removing trends resulted in the expected decrease in the correlation coefficient, suggesting that temperature sensitivity of Sphagnum growth is primarily influenced by seasonal temperature dynamics. Overall, our data indicate that a minor temperature fluctuation had a weaker effect on the growth rate (r = 0.23; n = 1439; P = 7.0 × 10^−19^). However, Sphagnum growth responded similarly to minor temperature fluctuations as described above along the K_p_ gradient, and comparable K_p_ ranges supported the growth response.

According to Figure 6, the K_p_ range less than 0.87 is characterized by the weakest growth response to minor temperature fluctuations (r = 0.17; n = 352; P = 1.5 × 10^−3^). The K_p_ range from 0.87 to 1.61 is where the perception of minor fluctuations of temperature, as well as temperature in general, is most favorable. Here, the growth response to temperature fluctuations (r = 0.33; n = 464; P = 2.3 × 10^−13^) was clearly above the significance level. However, in the range of K_p_ > 1.61, the growth response to minor temperature fluctuations (r = 0.19; n = 623; P = 3.2 × 10^−6^) is again seriously weakened.

Thus, the identified K_p_ range from 0.87 to 1.61 was maintained while examining the influence of minor fluctuations of temperature on growth rate fluctuations. This indicates that the revealed K_p_ interval is unlikely to be an artifact and likely reflects objective geomagnetic conditions that enhance the growth rate’s response to temperature changes.

### 3.5. Change in Growth Response to Diurnal Temperature along the K_p_ Gradient

**Figure 7:**
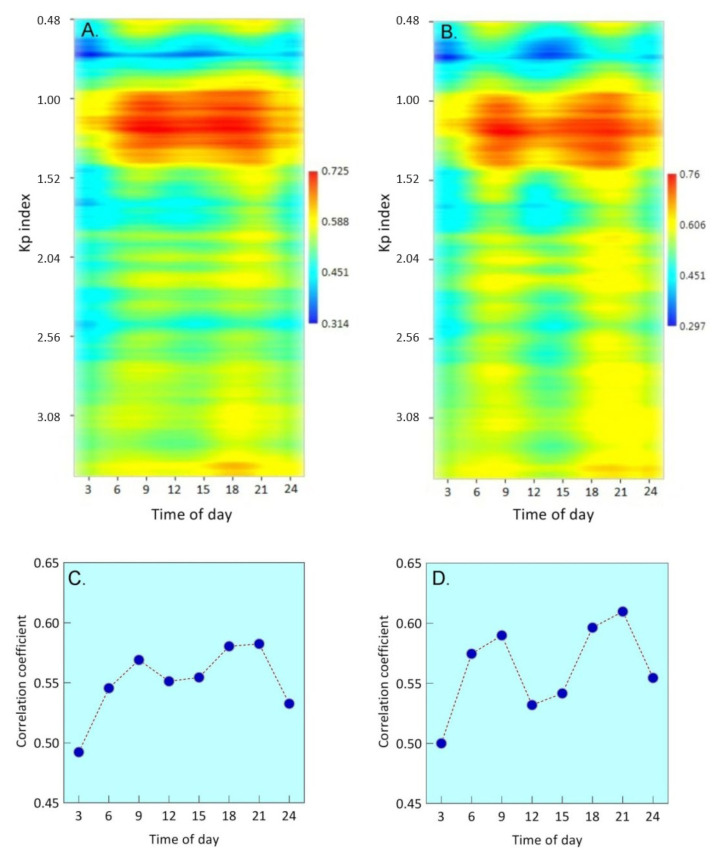
Change in the growth response of *S. riparium* to diurnal temperature along the K_p_ gradient. (**A**) Change in the growth response to the diurnal air temperature along the K_p_ gradient (the scale unit is the value of the correlation coefficient). (**B**) Change in the growth response to the diurnal soil surface temperature along the K_p_ gradient. (**C**) The growth response to diurnal air temperature on the full data set. (**D**) The growth response to diurnal soil surface temperature on the full data set.

In conclusion, we examined the change in the growth response of *S. riparium* to diurnal temperature along the K_p_ gradient. This analysis confirmed the presence of the favorable K_p_ interval described above, where Sphagnum exhibits the highest growth response to temperature. However, the most intriguing finding of this analysis was the differences in temperature response at different times of the day. The temperature growth response has two characteristic peaks, which are separated from each other by a decline (Figure 7). The first peak occurs between 6 and 9 h, and the second peak occurs between 18 and 21 h. A decline occurs between 12 and 15 h. Because these peaks may not be clearly noticeable with air temperature data (Figure 7A,C), we also involved soil surface temperature data (Figure 7B,D), which was recorded at the weather station. When utilizing this data, the peaks of the growth response intensify, and the decline between them deepens. Thus, our findings indicate that Sphagnum growth response to diurnal temperature follows a bimodal pattern. This bimodal pattern is noticed in both favorable and unfavorable geomagnetic conditions.

## 4. Discussion

Temperature is a crucial environmental factor that receives careful attention in plant studies [37,38]. However, the unresolved issues persist in understanding how plants perceive and translate it into their processes. The discoveries in recent years have seriously advanced our knowledge of temperature perception by plants [4,39]. Thus, it was shown that phytochrome B and phototropin photoreceptors can simultaneously act as thermoreceptors [8,9,11], which allow plants to more flexibly adapt to continuously changing temperatures. The molecular mechanisms underlying plant temperature responses are well understood [38,40], but there is still a gap in our knowledge regarding the ability of external factors to modulate temperature responses. Partially filling this gap, our study explores how the geomagnetic K_p_ index modulates the temperature growth response of *S. riparium*.

The importance of temperature is clearly emphasized by the fact that it is a clear zeitgeber for the growth of plants, including *S. riparium* [29]. This feature follows from the strong positive relationship between growth rate and temperature, which we estimated here to be r = 0.58 (n = 1439; P = 1.7 × 10^−119^). This estimate is similar to the one previously reported for the initial 4 years of monitoring [29], and the inclusion of 4 more years had minimal impact.

The relationship between growth and productivity of sphagnum mosses and temperature has been discussed in numerous papers [29,41,42,43,44,45,46,47,48,49], but not all of them obtained such high estimates of the temperature effect. One of the reasons is the coarser resolution of these studies, which does not allow registration of the growth limitation by low temperatures at the beginning and end of the growing season. Another reason is that under natural conditions, a temperature rise stimulates physiological processes in sphagnum mosses, while negatively impacting external moisture [50]. Therefore, its effect on growth and productivity is ultimately determined by the balance between these two components in a particular place of study. In our case, *S. riparium* grew in waterlogged habitats. Therefore, the temperature mainly affected physiological processes but rarely limited the external moisture of the sphagnum mat. The limitation was observed only in July–August in some years, when the mire water level strongly decreased under a combination of high temperature and a long absence of precipitation [29]; however, this process did not systematically affect the results of our study.

In contrast to temperature, we found no growth response of *S. riparium* to the geomagnetic K_p_ index. The initial growth rate (r = 5.4 × 10^−6^; n = 1439; P = 0.99) and its residuals (r = 0.03; n = 1439; P = 0.20) did not significantly correlate with the K_p_ index, suggesting that geomagnetic field variations did not directly affect Sphagnum growth during monitoring. The influence of magnetic fields on sphagnum mosses has not yet been studied, so our result is difficult to compare with other studies. In general, for plants, most studies were carried out in artificial strong magnetic fields, and their influence on the processes is reviewed in detail in recent articles [16,18,51]. At the same time, the sensitivity of plants to the geomagnetic field [22,52,53], unlike animals and humans [54], is still poorly understood.

The modulation of signaling and metabolic processes in plants by the geomagnetic field is the subject of a growing number of studies [22,55,56,57,58,59]. In the introduction, we presented the background that the temperature growth responses of plants can also be modulated by the geomagnetic field. The results of our study, which showed the selectivity of the temperature growth response of *S. riparium* to the geomagnetic K_p_ index, are consistent with this hypothesis. We observed the strongest response at 0.87 < K_p_ < 1.61, where the correlation between growth and temperature (r = 0.65; n = 464; P = 4.5 × 10^−58^) consistently exceeded the average value r = 0.58 (n = 1439; P = 1.7 × 10^−119^). There was a relatively weak response when K_p_ > 1.61 (r = 0.54; n = 623; P = 1.7×10^−48^); the correlation of growth with temperature in this case was below the average value. An analysis of the growth response to the temperature residuals and diurnal temperature showed similar K_p_ intervals, which additionally confirms the modulating effect of the geomagnetic K_p_ index on the temperature growth response of *S. riparium*.

Since the geomagnetic K_p_ index is affected by solar activity [28], which is also a weak growth factor for *S. riparium* [32], we do not exclude some contribution to the results. The cross-talk between UV-B photoreceptor UVR8 and phytochrome B signaling pathways provides the physiological basis for this view [60]. This crosstalk could potentially attenuate the temperature growth response of *S. riparium* at elevated UV-B levels, which corresponded to increased solar activity and an increased K_p_ index. Since variations in solar activity make a very small contribution to diurnal variations in ambient UV-B, their potential contribution to weakening the temperature sensitivity of Sphagnum growth along the K_p_ gradient appears to be very small. However, it seems that our data contradict this potential mechanism, since they show a weak tendency to increase (but not weaken) the growth response to temperature with an increase in the K_p_ index.

During the growth period, temperature is highly correlated with light, and both of these signals are integrated in phytochrome B-mediated responses [9]. Therefore, it may not seem obvious that the perception of temperature or light was more strongly modulated by the K_p_ index in our study. The results leave some uncertainty in this matter, but a number of indirect signs point to temperature. For example, the growth response pattern of *S. riparium* to temperature along the K_p_ gradient changed very little after seasonal trends were removed. At the same time, the magnitude of correlation coefficients changed slightly under favorable K_p_ conditions compared to the initial pattern. Thus, the modulating effect of the geomagnetic field on the temperature growth response was preserved regardless of the seasonal effect, which strongly combined the temperature and light levels. Also, K_p_ modulated the growth response to temperature not only in the daytime but also in the dark. This effect could be the result of a fundamental correlation between diurnal temperatures at different times, but we see that its severity is close between contrasting lighting conditions (for example, between 12 and 24 h). Finally, analysis of the change in the growth response to day length along the K_p_ gradient showed (unpublished data) that the optimal geomagnetic conditions for it are shifted towards a higher K_p_ index. Thus, these features together indicate that the patterns obtained were the result of modulation of the growth response to temperature rather than light.

During the study, we carried out monitoring in two nearby mires, but it is unlikely that this fact had a serious impact on the results of the study. Although the geomagnetic field in each of these mires could be slightly different for geological reasons, in this study, we considered variations in the geomagnetic field that should have been approximately the same and synchronous in both mires.

Our particular interest was related to the analysis of the influence of diurnal temperature on the growth rate of *S. riparium*, since there are still no data on this issue.As in previous analyses, a clear modulating effect of the K_p_ index on the growth response to temperature was found here. However, in addition to this, characteristic differences in the time of day were discovered. In particular, the range of favorable geomagnetic conditions for the growth response to temperature at 6–9 and 18–21 was wider, and the growth response there was stronger than at other times of the day. It is noteworthy that the clarity of this bimodal pattern depended on the temperature of the substance at the weather station we used in the analysis. When air temperature was used, the bimodal pattern was less clear than when soil surface temperature was used. This phenomenon seems paradoxical, since, despite the ability of air to mix and therefore better influence *S. riparium*, our data showed that the influence of soil surface temperature, which does not have a direct influence on the studied moss, was stronger. The reasons for this phenomenon are not entirely clear, but one explanation may be the presence of biological processes in soils that also have a similar bimodal pattern. If these processes produce even a small amount of heat, this may be enough to simultaneously raise or lower the soil temperature at the weather station and at sites remote from it. Similar bimodal patterns are known for plant photosynthetic productivity [61,62,63,64], which also increases in the morning and evening hours. They have also recently been identified in the respiration of Sphagnum-dominated peatlands and have been shown to be controlled by daylength [65]. It is noteworthy that around the hours when we observed the strongest effect of temperature on growth rate (6–9 and 18–21 h), dips in peatland respiration were observed. This suggests that the timing of the strongest growth response to Sphagnum temperature corresponded to the greatest photosynthetic productivity when CO_2_ was used for the growth process.

## 5. Conclusions

Temperature is considered to be one of the strongest and relatively constant factors for plant processes. However, our long-term observations of the growth of peat moss *Sphagnum riparium* in the natural environment showed that its effect on the growth rate depends on the geomagnetic field conditions. The strongest temperature influence was recorded at the geomagnetic K_p_ index from 0.87 to 1.61, and about a third of the days of the Sphagnum growing season fell within this range. Outside of this range, temperature clearly had a weaker effect on the growth rate of Sphagnum. Potentially, this phenomenon could be caused by the interaction of plant photo/thermoreceptors (phytochromes and phototropins) with cryptochrome photoreceptors, which are involved in the perception of the magnetic field in many organisms. The current study cannot confirm or refute this hypothesis, since this requires special studies under controlled conditions that will combine molecular and ecological approaches. However, regardless of how Sphagnum perceives the geomagnetic field, the discovered phenomenon may have a serious ecological perspective. If future studies confirm the widespread geomagnetic field dependence of temperature-sensitivity processes among plants, this will open up new horizons for our better understanding of species competition, distribution, and evolution.

## Figures and Tables

**Table 1:** The main parameters of *S. riparium* growth monitoring.

	2015	2016	2017	2018	2019	2020	2021	2022	2015–2022
Number of sampling events, days	34	68	88	89	77	102	92	90	640
Number of sample plots	4	11	13	6	3	10	10	10	3–13
Number of shoots measured	9087	30,267	45,278	31,837	10,526	34,195	24,000	24,300	209,490
Number of growth rates from sample plots	530	1365	1578	1020	544	1706	1608	1608	9959
Number of growth rates from mire area	178	178	178	180	156	205	184	180	1439
Mean sample size, shoots	93.7	59.7	57.8	60.0	46.0	39.9	30.0	30.0	52.1
Mean interval between sampling events, days	5.2	2.8	2.0	2.1	2.1	2.0	2.0	2.0	2.5

## Data Availability

The author declares that all data supporting the findings of this study are available in the paper.
